# Migration to breeding areas by male sperm whales *Physeter macrocephalus* from the Northeast Atlantic Arctic

**DOI:** 10.1038/s41598-025-91266-8

**Published:** 2025-03-06

**Authors:** Christian Lydersen, Marie-Anne Blanchet, Kit M. Kovacs, Tiu Similä, Carla Freitas, Zoë Morange, Ove M. Pedersen, Emma F. Vogel, Marten Bril, Guttorm Christensen, Audun H. Rikardsen

**Affiliations:** 1 Norwegian Polar Institute, Fram Centre, 9296 Tromsø, Norway; 2 Faculty of Biosciences, Fisheries and Economics, UiT – The Arctic University of Norway, 9019 Tromsø, Norway; 3 Whale2Sea, Hamnegata 9, 8480 Andenes, Norway; 4 Institute for Marine Research, Flødevigen Research Station, 4817 His, Norway; 5 MARE-Marine and Environmental Sciences Centre, 9020-105 Funchal, Madeira, Portugal; 6 Akvaplan-Niva, Fram Centre, 9296 Tromsø, Norway; 7 Norwegian Institute of Nature Research, Fram Centre, 9296 Tromsø, Norway

**Keywords:** Distribution, Sexual segregation, Year-round midlatitude breeding, Ecology, Zoology

## Abstract

Mature male sperm whales (*Physeter macrocephalus*) primarily inhabit high latitude regions, travelling to tropical/temperate waters for breeding, where females and juveniles reside in cohesive social groups. Though mating is known to occur at low latitudes, the timing, duration, and routes of adult male migrations between feeding and breeding areas are poorly known. To study movement patterns of adult male sperm whales, 29 individuals were equipped with satellite transmitters in the Northeast Atlantic Arctic (69–79°N). Twelve of these animals undertook southward migrations. Departures from northern latitudes occurred asynchronously from January to October, indicating that sperm whales do not have a well-defined breeding season. Migrating males travelled 40 (± 11) d to reach the breeding areas at latitudes below 45°N. They travelled distances of 3,993–7,951 km. They spent 76 (± 22) d in the south, roaming across an enormous region (˃10 million km^2^). Dives deeper than 1,000 m occurred both during migration and at the breeding grounds. Two whales were tracked back to Arctic waters. Their trips took 175 and 180 d, with cumulative distances travelled being 16,332 km and 17,669 km, respectively. This study fills important knowledge gaps in the annual cycle of these cosmopolitan giants.

## Introduction

Sperm whales (*Physeter macrocephalus*) are one of the most widely distributed cetaceans; they can be found throughout the worlds’ oceans from the pack-ice edges at high latitudes to tropical waters^1^. The species exhibits an extreme sexual dimorphism, with males reaching up to 18 m long and weighing up 60 tons, while the corresponding values for females are 11 m and 25 tons^2^. Only mature males range into polar regions, while females and younger animals occupy tropical and midlatitudes waters where they live in coherent social groups^2^. This species was exposed to heavy commercial exploration, which ceased only at the end of the 1980ies. The sperm whale global population size is currently estimated to be circa 850,000, down from an estimated pre-whaling abundance of about 1,9 million animals^3^.

Given the large numbers and wide distribution of sperm whales, there is surprisingly little knowledge about their general ecology, migration patterns, and the breeding seasons of these animals, especially related to males that spend much of the year at high latitudes. Based on recovery of old harpoons and discovery tags and photographic matches between areas, males are known to travel long distances between high latitudes and low latitudes, while females generally remain in warmer midlatitude waters^4–7^. However, detailed movement data is scarce. Most biologging studies conducted on adult male sperm whales have involved short-term deployments of instruments that have provided detailed information on diving, foraging, interactions with fishing gear and other types of behaviour^8–16^. A few satellite-tracking studies have been conducted at low latitudes^17–21^, and some few adult males have been tagged in the northern hemisphere that have shed light on movements and migrations from high to low latitudes in the Pacific Arctic and the Northwest Atlantic^22–24^.

In the North Atlantic several mark-recapture studies have shown connectivity between sperm whales across ocean basins: Azores – Spain^6^, Canada – Spain^4^, Gulf of Mexico – Azores, Iceland – Azores^5^, northern Norway – Azores^7^. However, there is virtually no information about migration routes or the phenology of migration in the North Atlantic. Also, very little is known about the seasonality of breeding, which is known to take place at low latitudes. It has been suggested that sperm whales can mate through most of the year, although March–June is thought to be the peak period in the Northern Hemisphere^2^. Only one study from the North Atlantic has tracked a single male sperm whale from its northern feeding grounds (Davis Strait in Arctic Canada ~ 68°N) south to Bermuda and beyond—a trip of more than 5,500 km. Given that the whale was still in transiting mode when tag transmissions ceased, it is likely that it had not yet reached its destination^23^.

In the Northeast Atlantic Arctic, sperm whales are found mainly in deep basins in the Norwegian Sea^25^. However, they also occur along the shelf breaks off the coast of northern Norway and west of Svalbard^25–28^. In this latter region sperm whales can be found above 80 N°^26,29^. The most recent abundance estimate for sperm whales in the Northeast Atlantic region is about 5,700 animals^25^. However, the timing of migrations to their lower latitude breeding areas and where these areas are situated are unknowns. Thus, the present study explores movement patterns of adult male sperm whales instrumented with satellite-tracking devices in Arctic areas of the Northeast Atlantic. The specific objectives of this study were to document migration routes and phenology and to identify their breeding areas.

## Material and methods

### Tagging procedures and data collection

**Fig. 1:**
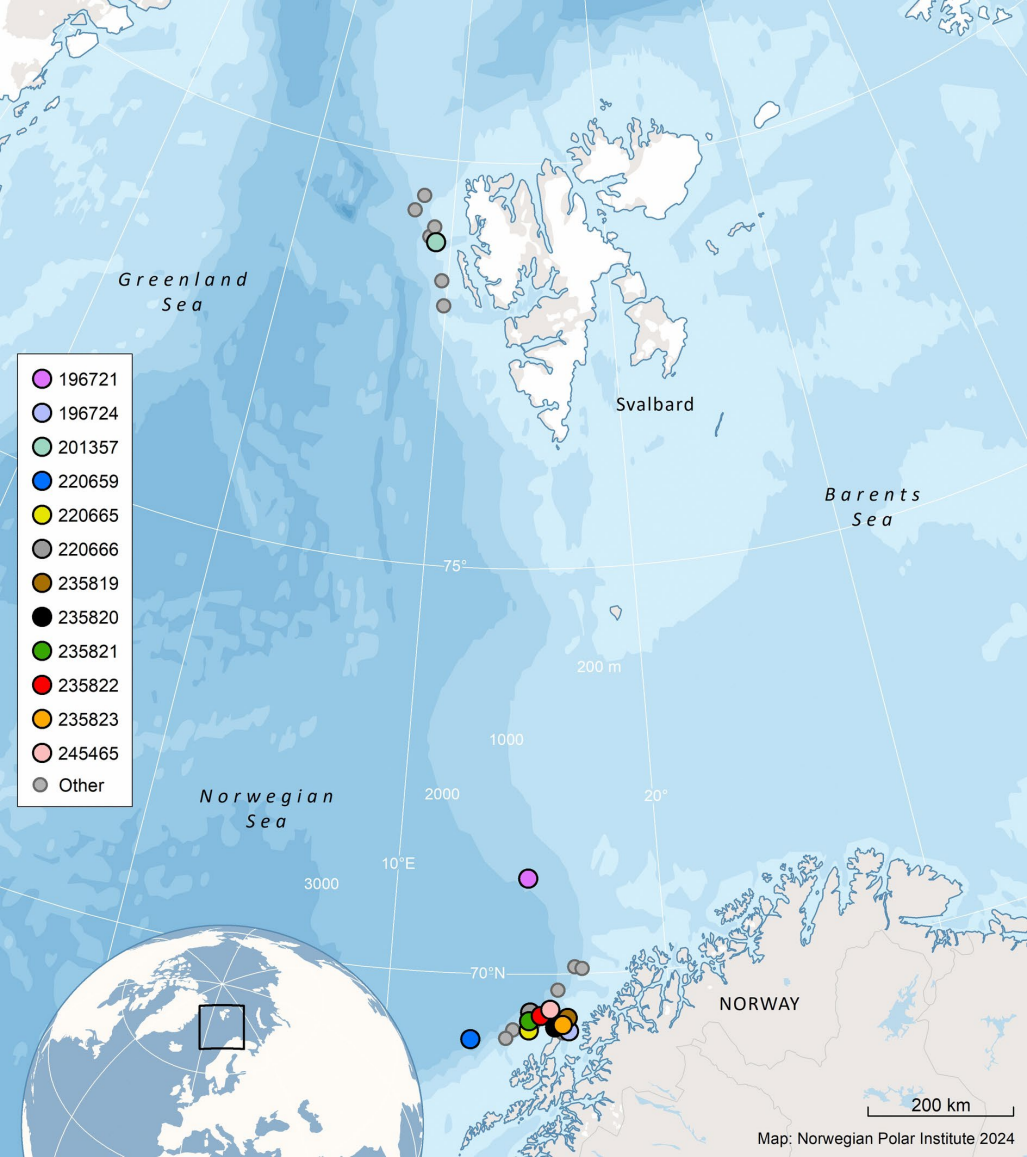
Tagging locations for 26 adult male sperm whales equipped with satellite tags in Arctic Norway, 2020–2023. Tagging locations of animals that did not migrate (N = 14) are symbolized with small grey circles. Tagging locations of the animals that did migrate (N = 12) each have a unique coloured circle. The map was generated based on publicly available ArcMap polar projections documents using ArcGIS 10.1 (www.esri.com).

Twenty-nine adult male sperm whales were tagged between 01 August 2020 and 10 June 2023 along the continental shelf edge west of northern Norway (n = 22) and around Svalbard (n = 7), between 69.2°N and 79.4°N (Fig. 1, Table 1). The whales were instrumented with either Wildlife Computer SPOT-303/372 or SPLASH-10302/373 transdermal tags (https://wildlifecomputers.com).

**Table 1:** Summary data for deployments of satellite tags on 26 adult male sperm whales from Arctic Norway. Mean time step refers to the average time between locations.

Whale ID	Tagging location lat., long	Tag type	Tagging date	First location	Last location	Track duration (d)	Number of days with location	Total number of locations	Mean time step whole track (h)	Mean time step segments (h)	Max gap duration (d)	Number of gaps > 72 h	Number of segments	Mean segment duration (d)
196,721	72.0°N,15.9°E	SPOT	3-Aug-20	3-Aug-20	24-Mar-21	233	225	2254	2.50	2.41	5	2	3	75
196,722	70.2°N,17.0°E	SPOT	6-Aug-20	6-Aug-20	15-Oct-20	70	65	566	2.95	2.68	7	1	2	32
196,723	69.8°N,16.5°E	SPOT	1-Aug-20	1-Aug-20	4-Dec-20	125	126	1571	1.90	1.90	1	0	1	124
196,718	78.8°N,08.5°E	SPOT	22-Sep-20	22-Sep-20	9-Jan-21	109	110	3760	0.68	0.68	0	0	1	109
201,347	79.4°N, 07.6°E	SPLASH	19-Sep-20	19-Sep-20	2-Nov-20	44	44	385	2.68	2.68	2	0	1	43
201,353	79.2°N,08.3°E	SPLASH	19-Sep-20	19-Sep-20	26-Nov-20	68	69	631	2.64	2.64	1	0	1	68
201,354	78.5°N,08.5°E	SPLASH	22-Sep-20	22-Sep-20	8-Nov-20	47	48	250	4.54	4.54	1	0	1	47
201,357	79.1°N,08.4°E	SPLASH	19-Sep-20	19-Sep-20	6-Apr-21	199	136	1375	3.53	2.40	51	2	3	66
201,358	78.8°N,08.7°E	SPLASH	18-Sep-20	18-Sep-20	2-Dec-20	75	76	767	2.38	2.38	1	0	1	75
201,360	78.2°N,10.4°E	SPLASH	22-Sep-20	23-Sep-20	5-Nov-20	43	44	254	4.13	4.13	1	0	1	43
196,720	69.4°N,16.3°E	SPOT	14-Mar-21	14-Mar-21	27-Oct-21	227	228	3278	1.66	1.66	1	0	1	226
196,724	69.4°N,16.3°E	SPOT	14-Mar-21	14-Mar-21	13-May-21	60	60	1012	1.47	1.47	2	0	1	60
220,658	69.4°N,16.1°E	SPLASH	6-Jun-21	6-Jun-21	24-Jul-21	48	48	424	2.71	2.71	2	0	1	48
220,659	69.3°N,14.7°E	SPLASH	11-Aug-21	11-Aug-21	15-May-22	277	200	609	10.97	9.10	11	10	11	28
220,661	69.4°N,15.3°E	SPLASH	11-Aug-21	11-Aug-21	28-Oct-21	78	46	338	5.53	3.27	32	1	2	23
220,664	69.4°N,15.2°E	SPLASH	5-Jun-21	5-Jun-21	13-Jun-21	8	9	54	3.40	3.40	1	0	1	8
220,665	69.3°N,16.1°E	SPOT	6-Jun-21	6-Jun-21	15-Nov-21	162	135	1380	2.84	2.38	18	3	4	46
220,666	69.4°N,15.1°E	SPOT	5-Jun-21	5-Jun-21	5-Aug-21	61	62	672	2.19	2.19	1	0	1	60
220,662	69.2°N,14.2°E	SPLASH	31-May-22	1-Jun-22	13-Jul-22	42	41	286	3.55	3.55	3	0	1	42
235,821	72.0°N,15.9°E	SPOT	24-Aug-22	24-Aug-22	23-Mar-23	211	208	1932	2.67	2.67	3	0	1	211
235,822	70.1°N,16.9°E	SPOT	25-Aug-22	25-Aug-22	5-Apr-23	223	193	1968	2.73	2.37	30	1	2	97
235,819	69.4°N,16.2°E	SPOT	27-Mar-23	27-Mar-23	7-Jun-23	72	70	298	5.80	5.56	3	1	2	68
235,820	69.4°N,16.2°E	SPLASH	27-Mar-23	27-Mar-23	23-Oct-23	210	152	776	6.49	5.36	6	9	10	24
235,823	69.3°N,16.3°E	SPOT	26-Mar-23	26-Mar-23	7-Nov-23	226	227	2314	2.36	2.36	1	0	1	226
235,824	69.5°N,15.4°E	SPOT	10-Jun-23	10-Jun-23	26-Oct-23	138	138	2397	1.45	1.45	1	0	1	138
245,465	69.4°N,15.3°E	SPLASH	10-Jun-23	10-Jun-23	24-Jul-23	44	45	454	2.35	2.35	1	0	1	44

Some of the tags used in this study were modified slightly, including custom-ordered stiffer petal blades to potentially increase the tag retention time. The attachment rings were also slightly altered to make larger drainage holes between the tag and the attachment ring, so that water drained away from the dry sensor more easily. These adjustments are now standard on the tags produced by the manufacture. Additionally, extra cutting blades were welded on the spring-loaded anchors on some of the 302/303-version tags to potentially reduce the penetration force (less power on the air gun) needed to get through the hard skin and blubber of the sperm whales (note: original anchors were removed from the tags during welding to avoid heating of the electronics).

All tags were deployed using a modified Air Rocket Transmitter System (ARTS^30^) at distances between 3–12 m using ~ 20 bar pressure. For the 22 whales tagged along the continental shelf edge of northern Norway we used one of two types of boats to approach the whales; large RIB-boats (26–36 ft) with outboard engines starting from a land base, or with a waterjet RIB-boat (26 ft) launched from a Norwegian Coast Guard vessel. Both types of boats had a purpose-made platform placed at the bow that gave extra height and support for the person doing the tagging. The tag was usually launched at a downward angle, targeting an area close to the base of the dorsal fin or as high up as possible in the area in front of the dorsal fin. The whales were approached slowly at a slight oblique angle from behind until an approximately 90° angle to the side of the whale was achieved. For the seven whales tagged at the continental shelf edge off the west coast of Svalbard we used a helicopter (Eurocopter 350 Ecureuil) carried onboard a ship. When a whale was spotted from the ship, the helicopter took off, approaching the whale from behind at low altitude with the tagger perched on the seat and outside struts on the right side of the helicopter behind the pilot. An area on the back immediately in front of the dorsal fin was targeted.

Animal handling protocols were approved by the Norwegian Animal Research Authority (FOTS permits ref: 20/24853, 20(98217 and 22/147871) and the Governor of Svalbard (permits refs: 20/00920-2). The research was carried out in accordance with the relevant guidelines and regulations. We have conducted and reported the research according to the ARRIVE guidelines relevant for research on wild animals.

The Wildlife Computer SPOT/SPLASH tags report the surface position of the whale while the SPLASH tags additionally report diving information. Both SPOT and SPLASH tags from Wildlife Computers transmit to the ARGOS satellite system. Consecutive transmissions received in a single satellite pass are used to calculate the location of the tag using the doppler shift method. Both tag types included 1.5 AA-lithium batteries together with the electronics in a stainless-steel casing (tube diameter: 24 mm), with a stainless-steel stop plate at the distal end to prevent the tag from penetrating too deeply into the blubber. The stainless-steel tube was attached to an anchoring spear equipped with a sharp tip and foldable barbs along the length of the spear. The total length of the tag from the stop plate to the tip was 300 mm and the weight of the instrument was 390 g. The two tag types deployed along the Norwegian mainland were programmed slightly differently. The Spot-tags were programmed to send about 20 transmission every h for the first four months, then reduce transmission rate to 8–12 transmissions/h so that the tag had battery power to send positions for about one year. The SPLASH tags were programmed to send 200 transmissions per day for the first 3–4 months (starting at midnight GMT and then every 2–4 h thereafter until all transmission for that day were completed) then reduce the transmission rate per month so that the tag should be able to send for up to a year. The SPLASH tags deployed in Svalbard were programmed to make 250 transmissions per day starting at 0600 GMT. All of the tags collected and transmitted information via the Argos satellite system (for details; see^31^). Location data were processed with the Kalman Filter by CLS Argos^32^. ARGOS locations were classified according to their accuracy; accuracy decreases in the following order 3,2,1,0,A,B. Location quality Z are those for which the location process failed; these Z class locations were removed prior to further analysis. All data processing and statistical analyses were performed using ‘R’ (version 4.1.1^33^).

### Track filtering and path reconstructions

To account for location uncertainty of the Argos positions, and the irregular timing of positions received from the tags due to satellite availability and the surfacing behaviour of the whales, locations were filtered, modelled, and then predicted at regular time intervals of 2 h. We first adjusted duplicated time stamps by adding 1 s using the function “adjust.duplicateTimes” in the package “trip”^34^. Locations were then filtered with a speed, distance and angle filter using a 5 ms^−1^ threshold and the default values for the angles and distances^35^. We then used a continuous-time state-space model fitted with a simple random walk using the “fit_ssm” function from the “aniMotum” package^36^. Large gaps in tracking data can be problematic when fitting these models and particularly when estimating behavioural indices. We therefore fitted one model per individual on two datasets, one considering each track as a whole (including all gaps) and the other considering only track segments, with a minimum total duration of 4 d^37^ and at least one position per day. We used the first dataset to calculate track metrics such as total and cumulative distances, turning angles and timing and duration of migration. The second dataset was used to explore the whales’ behaviour and avoid making inferences in periods for which there were no data.

### Movement metrics

The following movement metrics were calculated to identify behavioural changes along individual migratory paths: speed, bearing, move persistence and time in area. Speed was defined as the average 2-h horizontal displacement between two consecutive modelled locations. It was calculated based on the Euclidean distance between two consecutive points on the 2 h interpolated dataset using only segments to avoid underestimating the real speed of the whales. It must be highlighted that since the whales do not necessarily move in straight line between these points, and since they spend most of their time underwater moving through a 3D environment, the distance (and hence speed) between two locations is underestimated. However, the cumulative distance was calculated over the entire track. The distance between two consecutive segments was measured as a linear interpolation, hence the true cumulative distance was underestimated. The maximum distances between the location of the start of the migration and the point of the track furthest away were calculated using the great circle distances. The bearings during the southbound leg of the migration were calculated between the location of the start of the migration and each subsequent position until the whale arrived on the breeding grounds (see definition below).

Move persistence (γt) is a continuous behavioural index that captures autocorrelation in both speed and direction^38^. Values range between 0 and 1, where low persistence (close to 0) designates highly variable movement patterns typically in a restricted area, and high persistence (close to 1) denotes consistent and directed movement^38^. Move persistence was estimated from locations estimated at 2-h intervals on segments using the ‘fit_mpm’ function in the ‘aniMotum’ package^36^. The move persistence model was fitted with a single pooled random variance parameter to facilitate comparison between individuals.

Time spent in area (TSA) was calculated using the tripGrid function in the trip R package^34,39^. Time spent (h) within a 50 × 50 km grid cell was calculated for each migrating individual using the 2 h interpolated dataset on segments to compare the whale’s area use between their resident and migratory phases.

### Migration and phenology

The start of the migration (hereafter named N-S transit) from the northern foraging areas, was determined in two steps. First, we defined migratory and non-migratory individuals based on the bimodal distribution of each individual’s maximum distance from the tagging location calculated as the great circle distance (Fig. S1a). Non-migratory individuals moved less than 1,000 km from the tagging location. We then delineated a foraging polygon based on the tracks of these non-migrating individuals (that only occupied the foraging area during their tracking periods) using one location every other day to decrease the autocorrelation between consecutive points. We used the minimum convex polygon (MCP) method and defined the 100% contour as the foraging polygon (Fig. S2). The migratory phase was estimated to start when an individual left the foraging polygon (= the northern foraging grounds) and did not return to it (except if returning from the southern breeding grounds, a return hereafter called the S–N transit). Arrival at the area hereafter referred to as the breeding area was defined using two criteria 1) the geographic delineation by Rice^2^ that suggested that breeding areas are in the North Atlantic below 45°N and 2) a change in each whale’s movement patterns, assuming that a whale arriving to an area of interest would reduce its speed. We calculated the whales’ speed, then smoothed the curves using a local regression (loess function in the R package “stats” with a degree of smoothing α = 0.2). We then calculated the first order derivative on this curve to calculate the acceleration rate(s) over the tracks. We defined the “arrival” to the breeding area as the first point south of 45° N where an individual was decelerating (negative acceleration) (Fig. S3). Because few individuals had tracking durations long enough to again leave the breeding areas, and transmission rates were low towards the end of tracking periods, combined with poorer satellite coverage at lower latitudes, we defined the “departure” from the breeding area as being when an animal moved north of the average latitude defined above as entering the breeding area. The size of the breeding area was calculated as the 100% MCP including all locations collected after the arrival date in the breeding area and before the departure date.

### Diving

Only the SPLASH tags (N = 13) collected dive information. Four of these tags were deployed on whales that undertook migrations during the period the tags were transmitting data. These instruments store and transmit dive data in two main modes: dive data (time at depth TAD, maximum depth, dive duration) aggregated in 6 h periods (a maximum of four per day) and single random dives or depth readings. For each 6 h period the tag records the number of dives performed within each depth and duration bin and the percentage of time spent within a depth bin (TAD). We used two settings: whale ID 201357 was programmed with 14 depth bins with limits between bins of 2, 4, 10, 20, 40, 60, 80, 100, 150, 200, 250, 300, 350, > 350 m and 14 duration bin with limits between bins of 6, 8, 10, 12, 14, 16, 18, 20, 22, 24, 26, 28, 30 > 30 min. Dives shallower than 2 m and shorter than 1 min were ignored. The three other whales (IDs 220659, 235820, 245465) were programmed with the following set up: 10 depth bins with limits between bins of 50, 100, 200, 300, 500, 700, 1000, 1500, 2000, > 2000 m and seven dive duration bins with limits between bins of 10, 20, 30, 50, 60, 80, > 80 min. Dives shallower than 50 m and shorter than 5 min were ignored. In addition to the summary bins, a random selection of single dives is transmitted providing the depth, duration, and time stamp. The maximum depth reached per day is also reported.

Comparison of dive depth and duration between the various migratory phases (in feeding area, N-S migration, in breeding area, and S–N migration) were done using linear mixed effects models (LME) with animal ID as a random effect. Comparison with the null model was done by likelihood ratio tests (LRT). Maximum depth was log transformed to meet the assumptions of the model. Post hoc Tukey tests were run for each pairwise combination of migration stage to assess the differences between each stage.

### Modelling of movement metrics

We explored movement metrics in relation to the migration departure date. For this we used the 2-h interpolated dataset on segments using only individuals that migrated (n = 12). Movement metrics (move persistence, rate of change in latitude and longitude) were investigated separately as a function of the number of days to the start of the migration (negative day values were assigned prior to the departure date and positive values were assigned after the start of the migration). We used Generalized additive mixed models (GAMMs) with a Gaussian link, using the “gamm” function from the “mgcv” package^40^. We included number of days in relation to the migration departure date as a smooth term. Individual ID was included as a random effect, and as a grouping factor, in the temporal autocorrelation structure of the order 1 (corAR1) term. The model with move persistence as the response variable did not include an autocorrelation term because move persistence is itself a measure of autocorrelation. Move persistence was log transformed to meet the model’s assumptions. We used Akaike’s information criterion to select the best fit models. If two models had a Δ AIC < 2, the simplest one was chosen. To explore if the whales’ space use changed during the various parts of their feeding-migration-breeding cycle, we modelled TSA as a function of latitude and longitude. Because we were interested mainly in where the whales spent most of their time, we used a quantile regression using the 75th percentile. We used the R package “qgam” and the function qgam, which fits non-parametric quantile regressions, using TSA as a response variable and latitude and longitude as explanatory variables. This modelling framework is not implemented for mixed effects, so the ID of the animals was included as a fixed term. The predictions were then averaged across all individuals.

## Results

### Tracks

Location data were obtained from 26 of the 29 male sperm whales tagged off the continental shelf of Norway and Svalbard (Fig. 1). Whales were tracked for 8 to 277 d, providing a total of 30,005 locations (Table 1). Detailed information on tag performance is provided in Table 1.

#### Migration from the northern feeding grounds

**Fig. 2:**
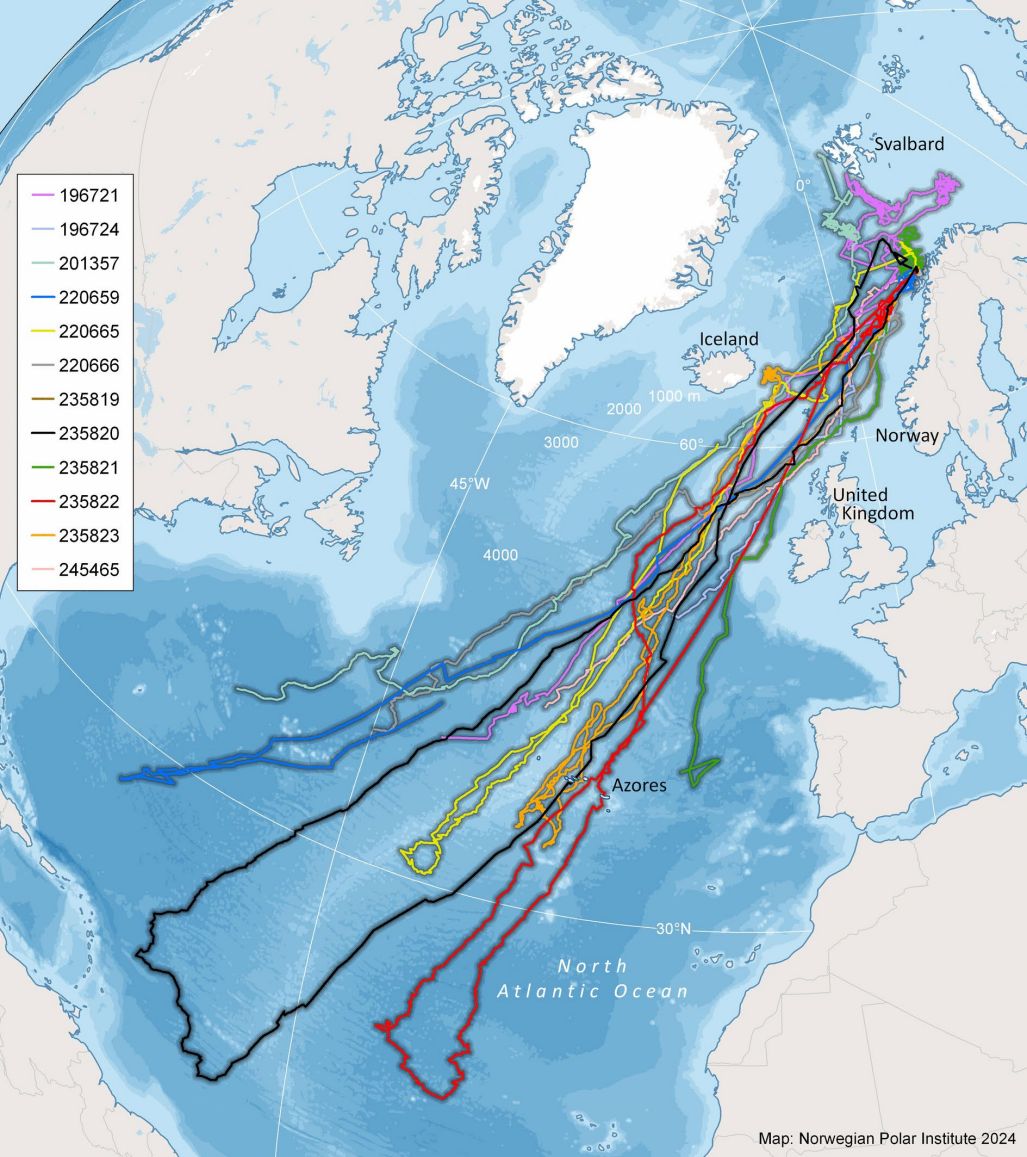
Modelled tracks for 26 adult male sperm whales, equipped with satellite tags in Arctic Norway (2020–2023). Tracks from animals that migrated (N = 12) each have a unique colour. The tracks are based on locations estimated ever 2 h by a state-space model. The map was generated based on publicly available ArcMap polar projections documents using ArcGIS 10.1 (www.esri.com).

Twelve of the 26 whales that reported data undertook migrations, leaving the northern foraging areas along the continental shelf between Svalbard and northern Norway to travel to breeding areas at lower latitudes (Fig. 2, Table 2). The 14 whales that remained in the northern foraging area throughout their tracking periods transmitted location data for an average of 80 ± 55 d (median 69 d, range 8–226 d, Fig. S1b). Data from these 14 whales are excluded from the further analyses in the current study. The average tracking duration for the 12 migrating whales was 165 ± 82 d (median 204 d, range 44–277 d. Estimating positions along the modelled tracks of the migrating individuals yielded n = 23,750 2-h interpolated locations.

**Table 2:** Summary information for breeding migrations of 12 adult male sperm whales instrumented with satellite tags at their feeding grounds in Arctic Norway. Not all individuals were tracked for their entire migration cycles. NA indicates where data is lacking for various parameters.

Whale ID	Tagging date	Start migration N-S	Arriving breeding area	Departing breeding area	Return to northern feeding ground	Duration N-S migration (d)	Time spent in breeding area (d)	Duration S–N migration (d)	Duration whole migration cycle (d)	Cumulative distance N-S (km)	Cumulative distance breeding area (km)	Cumulative distance S–N (km)	Cumulative distance whole breeding cycle (km)	Max distance from tagging location (km)	Total track duration (d)
196,721	3-Aug-20	03.feb.21	03.mar.21	NA	NA	28	NA	NA	NA	4,249	0	0	NA	4,745	233
196,724	14-Mar-21	21.apr.21	NA	NA	NA	NA	NA	NA	NA	0	0	0	NA	3,007	60
201,357	19-Sep-20	26.jan.21	03.mar.21	NA	NA	36	NA	NA	NA	4,470	0	0	NA	5,560	199
220,659	11-Aug-21	18.jan.22	18.feb.22	NA	NA	31	NA	NA	NA	3,869	0	0	NA	6,755	277
220,665	6-Jun-21	10.jul.21	01.sep.21	22.okt.21	NA	53	51	NA	NA	4,627	4,397	0	NA	5,494	162
220,666	5-Jun-21	09.jun.21	20.jul.21	NA	NA	41	NA	NA	NA	4,886	0	0	NA	4,934	61
235,819	27-Mar-23	01.jun.23	NA	NA	NA	NA	NA	NA	NA	0	0	0	NA	1,010	72
235,820	27-Mar-23	04.apr.23	13.mai.23	23.aug.23	26.sep.23	39	102	34	175	4,575	9,148	3,945	17,669	7,951	210
235,821	24-Aug-22	09.feb.23	12.mar.23	NA	NA	31	NA	NA	NA	3,634	0	0	NA	3,920	211
235,822	25-Aug-22	06.okt.22	08.des.22	02.mar.23	04.apr.23	63	84	33	180	5,877	7,005	3,449	16,332	6,788	223
235,823	26-Mar-23	05.apr.23	15.mai.23	22.jul.23	NA	40	68	NA	NA	4,426	5,169	0	NA	4,856	226
245,465	10-Jun-23	17.jun.23	22.jul.23	NA	NA	35	NA	NA	NA	4,181	0	0	NA	3,993	44

**Fig. 3:**
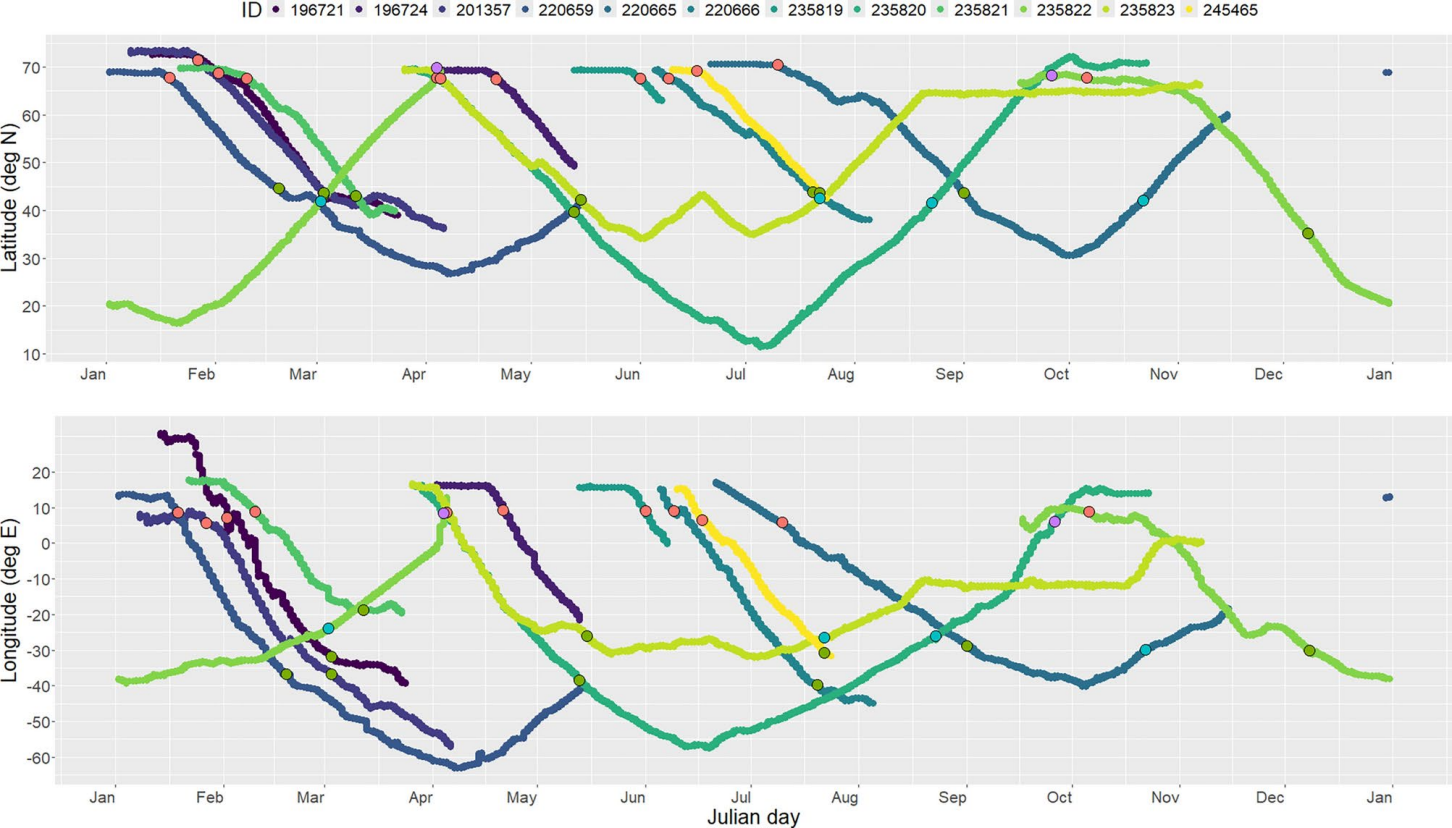
Changes in latitude and longitude during migration (starting 20 days before estimated departure) for 12 adult male sperm whales equipped with satellite tags in Arctic Norway, 2020–2023. X-axis represents the Julian day from the 1st of January. Red circles: estimated start date for the north–south part of the migration; green circles: arrival to the breeding area; blue circles: departure from the breeding area; purple circles: return to the northern feeding grounds.

Departures from the northern feeding grounds occurred asynchronously (Table 2, Fig. 3) between 18 January and 06 October, over a period that included 10 months of the year (261 d). All migrating individuals passed through the area between southeast Iceland and the Shetland Islands, on either side of the Faroe Islands (Fig. 2). The distribution of bearings for the N-S transit was centred around southwest for all individuals with little or no deviation (Fig. S4). The N-S transit lasted on average 40 ± 11 d (range 28–63 d, Table 2).

Two individuals (IDs 201,357 and 196,721) were tagged on 08 August and 19 September of 2020 on the edge of the continental shelf north of the Norwegian mainland and the northwest corner of Svalbard, respectively. These whales left the northern feeding ground 26 January 2021 and 03 February 2021, respectively. Their tracks were parallel with distances, at the closest, of 50 km initially, with a four day time difference. Subsequently, these two whales came together and migrated southward along the same path for about 300 km before separating once again. They both reached the southern breeding ground on the same day, 03 March 2021, but were 360 km apart upon their arrival.

#### Breeding area

After entering the breeding grounds (defined as south of 45° N), the distance between the 12 migrants’ tracks increased with the males spreading over an enormous, mainly offshore, area covering basically the whole longitudinal range of the southern part of the North Atlantic Ocean, from the ocean area off the Canary and Cape Verde Islands westwards to areas close to the Caribbean Islands and Brazil (Fig. 2). Three of the tagged males passed through the Azores, an area where sperm whales of all sex and age groups are observed year-round. However, none of them stopped in this area definitively, though one whale looped north and south several times, traversing the area four times immediately prior to the tag terminating its transmissions.

The distance from the start of migration to the furthest away locations at the breeding grounds was on average 5,500 ± 1,304 km (range 3,993–7,951; Table 2). The breeding grounds for the tracked sperm whales covered an area greater than 10 million km^2^. The four individuals that transmitted locations long enough to depart from the breeding area did so after spending 76 ± 22 d (range 51–102 d) in this southern region (Table 2).

#### Migration from the southern breeding grounds

All four individuals that had tags that transmitted locations for part, or all, of their S–N transit returned north through the same area between southeast Iceland and the Shetland Islands but not necessarily on the same side of the Faroe Islands that they swam through on the southbound journey (Fig. 2, Table 2). Two of the whales (ID 235820 and 235822) were tracked all the way back to the northern foraging grounds and thus provided complete migrations. The northbound transit for these two animals were accomplished in 34 and 33 days, respectively (Fig. 2, Table 1). The duration and cumulative distance covered by these two individuals were 175 d and 17,693 km and 180 d and 16,332 km, respectively. Another individual (ID 235823) was tracked on the S–N transit to the eastern edge of the Icelandic continental shelf (Fig. 2) where he spent nearly three months (20 July 2023 – 18 October 2023) in a very small area (100 km radius) presumably foraging. Connection was lost with this tag on the 07 November 2023; however, this animal was observed and identified based on photo-ID on 29 December 2023 (T. Simila pers. comm.), in the area where it had been tagged. The fourth individual that was tracked leaving the breeding area (ID 220665) stopped transmitting on 15 November 2021, 24 days after it left the breeding area. It was located just south of Iceland when data transmission ended.

### Surface behaviour

**Fig. 4:**
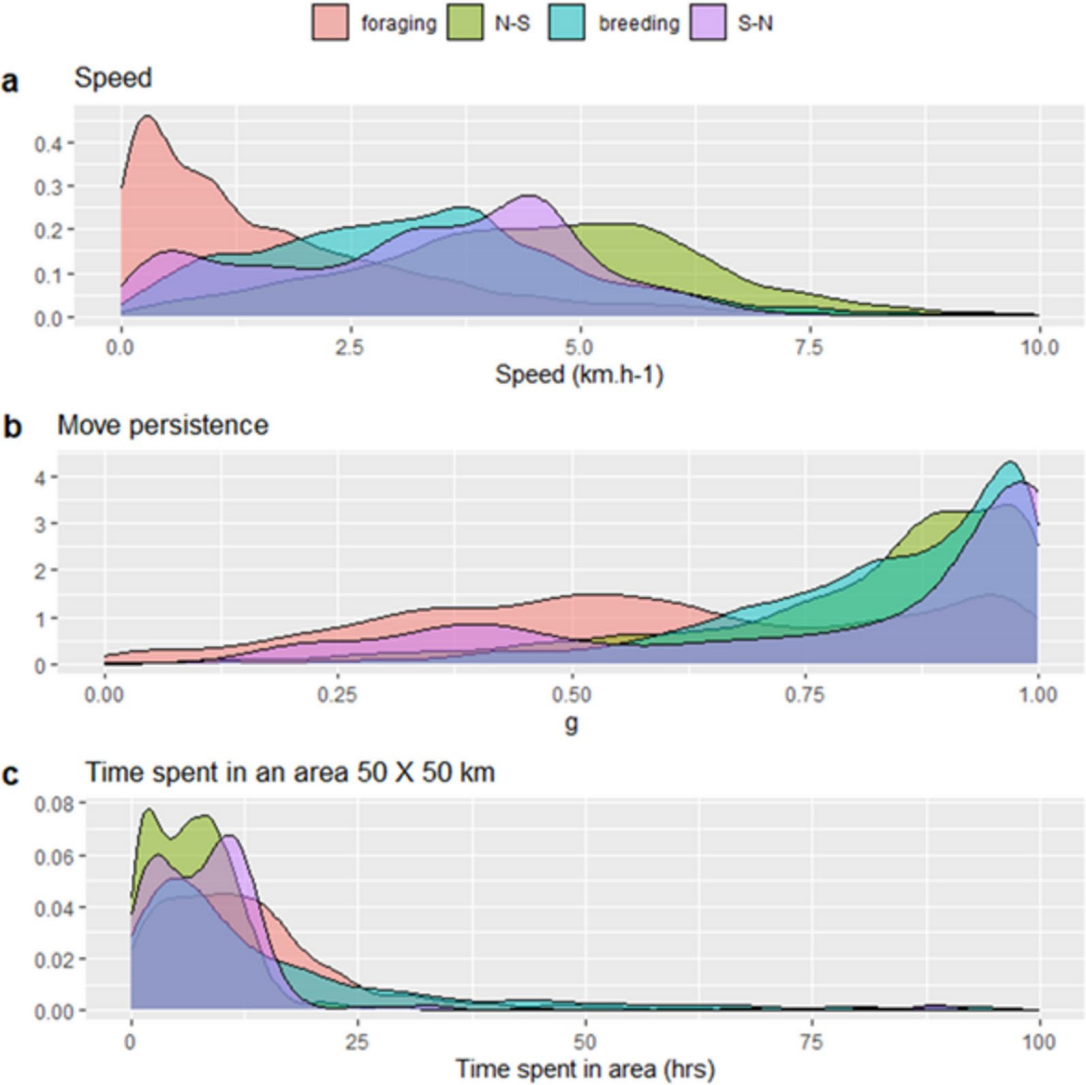
Distribution of the speed (**a**), move persistence values (**b**) and time spent in area (TSA) in a 50 X 50 km grid (**c**) for 12 adult male sperm whales equipped with satellite tags in Arctic Norway, 2020–2023. The distributions are colour-coded by the migration stage. Note that due to very long-tailed distributions the upper values of TSA were truncated at 100 h.

Surface behaviour of the whales varied between the different phases of their cycle. Particularly, movement metrics were different between the northern foraging areas vs when the whales migrated. In the northern feeding area, the whales swam slower (mean speed 2.0 ± 2.3 km h^−1^), within more restricted areas (low γ values, mean = 0.58 ± 0.25) and displayed higher TSA values (mean = 35 ± 92 h, Fig. 4) compared to the other migration phases. Once the whales started to migrate during their N-S or S–N transit, horizontal speed more than doubled compared to the northern foraging area (mean _N-S_ = 4.8 ± 2.5 km h^−1^, mean _S–N_ = 4.4 ± 1.4 km h^−1^). Their movements also showed strong consistent directionality characteristic of transit behaviour (high γ values mean _N-S_ = 0.79 ± 0.20, mean _S–N_ = 0.96 ± 0.1 km h^−1^). These movements were consistent with lower TSA values and were remarkably similar during the two transit phases (TSA mean _N-S_ = 7 ± 7 h; mean _S–N_ = 7 ± 4 h). Although the whales slowed down when arriving in the breeding area (mean = 3.5 ± 2.2 km h^−1^), they maintained a directionality similar to the transit phases (mean = 0.81 ± 0.18) but spent twice as much time in restricted areas (TSA mean = 13 ± 12 h) compared to transit phases.

The distribution of TSA was long tailed, with extreme values of up to 63 days during the foraging phase and up to 40 h during the migration phase estimated for individual whales. TSA also varied along the migration path with the highest values occurring in the breeding area (Fig. 4). ID 235823, that spent three months on the edge of the Icelandic continental shelf on its way back from the south, was excluded from this part of the analysis as an outlier (due to large numerical differences with the other individuals).

**Fig. 5:**
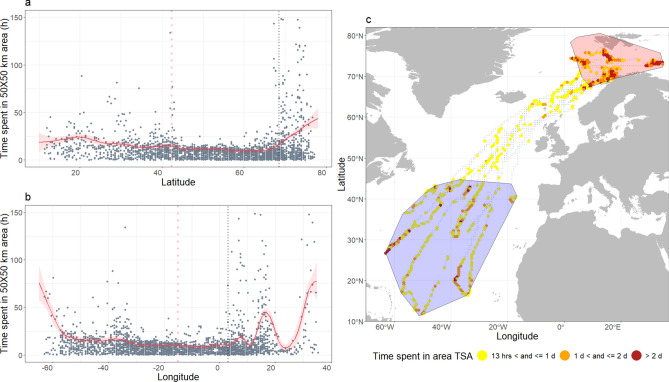
Output of a GAM quantile regression (75%) comparing time spent in area (TSA) on 50 X 50 km cells with latitude (**a**) and longitude (**b**) for 12 adult male sperm whales equipped with satellite tags in Arctic Norway, 2020–2023. Fitted estimates from the models (solid red curves) are plotted with the 95% CIs (red shaded areas) and over the data (grey dots) for the individual whales. The vertical black dotted line represents the southern latitude and western longitude extent of the northern feeding area polygon. The pink vertical dotted lines represent the southern and western limits of the breeding area polygon. The map (**c**) shows the same data in a spatial context with grey dots representing the centre of each 50 × 50 km cell along the tracks and the coloured dots the location of the 75th percentile of TSA values. The red and blue polygons show the limits of the feeding and breeding areas. The map was generated in Esri ArcMap v10.8.1, https://desktop.arcgis.com/en/arcmap/index.html.

**Fig. 6:**
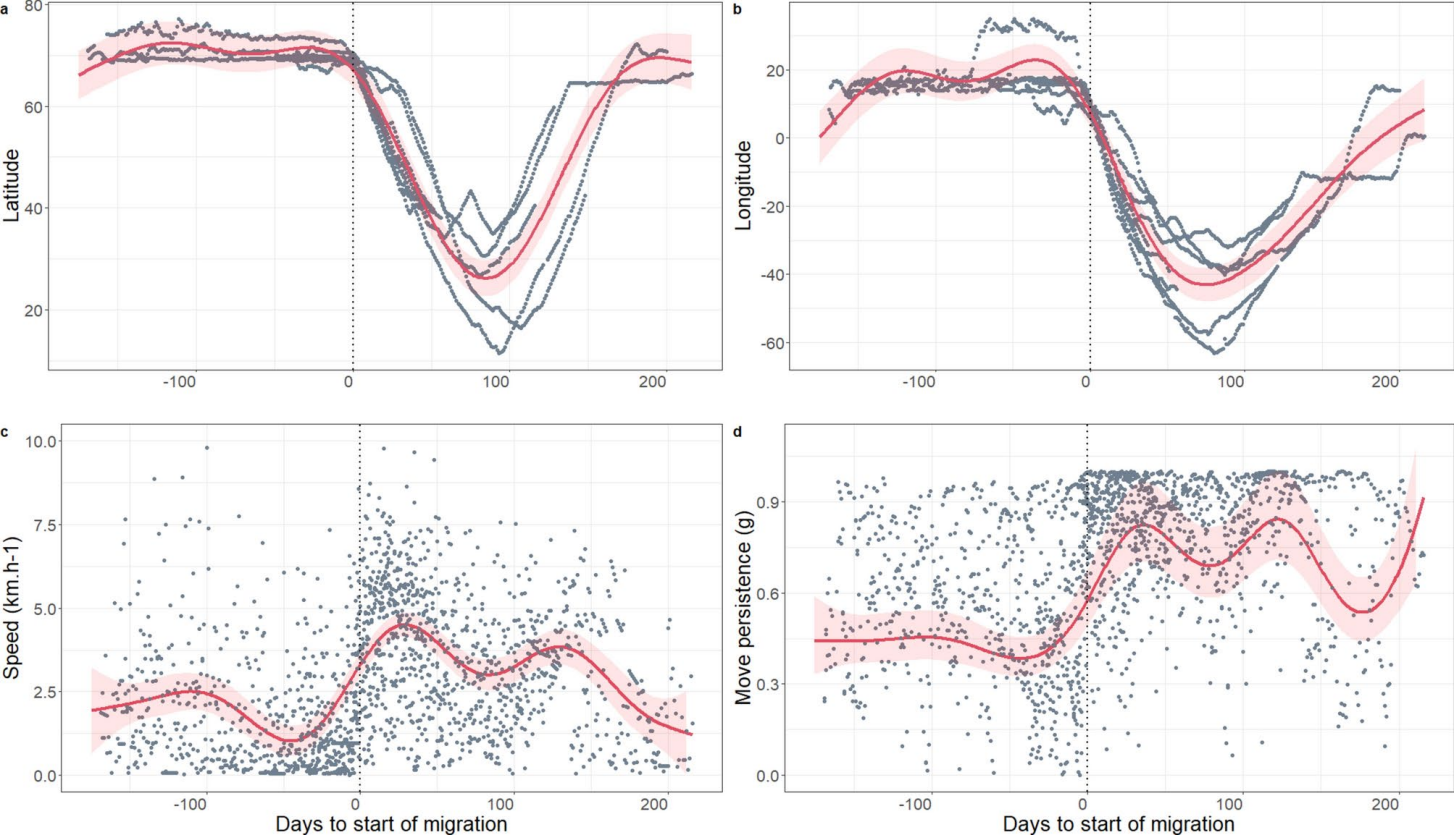
GAMM model outputs comparing (**a**) latitude, (**b**) longitude, (**c**) speed, and (**d**) move persistence as a function of the number of days before (negative numbers) and after (positive numbers) the start of the migration (vertical black dotted line) for 12 adult male sperm whales equipped with satellite tags in Arctic Norway, 2020–2023. Fitted estimates from the models (solid red curves) are plotted with the 95% CIs (red shaded areas) and over the data (grey dots) for the individual whales.

Despite a lack of synchronicity in migration phenology, movement patterns between whales were remarkably similar when the whales were in transit (Figs. 5, 6). The GAMMs showed that the start of the migration coincided with an increase in speed and of move persistence and a decrease in latitude and longitude, indicating a consistent and directed movement during the N-S and S–N transit phases (Table 3). Movement patterns were much more varied between the individuals after they reached the breeding area.

**Table 3:** Model structure, degrees of freedom and AIC values for models exploring movement (latitude (LAT), longitude (LON) and move persistence) for adult male sperm whales instrumented with satellite tags in Arctic Norway, relative to the number of days before migration from the northern feeding area. Bold text indicates the best-fit models.

	Model formula				
Movement metrics	Fixed effect	Random effect	Autocorrelation	df	AIC
LAT	~ s(day since start of migration)	random (1/ID)	corAR1(~ 1|ID)	6	15
	**~ s(day since start of migration)**	**random (1/ID)**	**corAR1()**	**6**	**15**
	~ 1			5	391
LON	~ s(day since start of migration)	random (1/ID)	corAR1(~ 1|ID)	6	441
	**~ s(day since start of migration)**	**random (1/ID)**	**corAR1()**	**6**	**441**
	~ 1			5	808
SPEED	~ s(day since start of migration)	random (1/ID)	corAR1(~ 1|ID)		3424
	**~ s(day since start of migration)**	**random (1/ID)**	**corAR1()**		**3424**
	~ 1				3426
MOVE PERSISTENCE	**~ s(day since start of migration)**	**random (1/ID)**		**5**	**223**
	~ 1			3	312

### Diving

Between 8 and 48% of the summary data, aggregated by 6 h periods were transmitted for the four migrating whales that had satellite tags that reported diving. The setting of the four tags were not identical and thus we report the diving results below in two groupings.

The whale with tag ID 201357 reported 2,891 dives, summarized in 6-h periods, for the 197 days of its tracking period. At least one 6 h period was recorded for each of 148 days. The number of dives in feeding/breeding areas and during the transit were nearly equally distributed with 1,339 and 1,397 dives (split between 746 during N-S transit and 806 in the breeding area), respectively. Most of the dives were deeper than 350 m (45%) and longer than 30 min (82%) and these values were similar between the breeding area and during transit. Information on maximum dive depths were available from only 63 days (not in the summary dive data stream). Dives deeper than 1,000 m were recorded both during transit and at the breeding grounds, with a maximum recorded depth of 1,344 m.

The three other whales equipped with satellite tags that recorded dive data collected a total of 2,055 dives, summarized in 6-h periods, split between the foraging area (n = 919), N-S transit (n = 778), in the breeding area (n = 317) and S–N transit (n = 41). Most of the dives lasted between 50 and 60 min (46%) and were shallower than 1000 m (70%). In the foraging area most of the dives were between 100 and 200 m whereas they were deeper during the transit phases (700 m–1,000 m) and in the breeding area (700 m–1,500 m). Maximum depth and duration were available for 1,054 individual dives equally split between the three individuals with SPLASH tags programmed the same way. The dives occurred in the foraging area (n = 475), N-S transit (n = 383), in the breeding area (n = 183) and the S–N transit (n = 13). The maximum recorded dive depth was 1,927 m; this dive lasted 53 min. The longest recorded dive was 68 min with this dive reaching a depth of 1191 m. Both of these record dives were performed by the same individual (whale ID-235820), when it was in the breeding area.

**Fig. 7:**
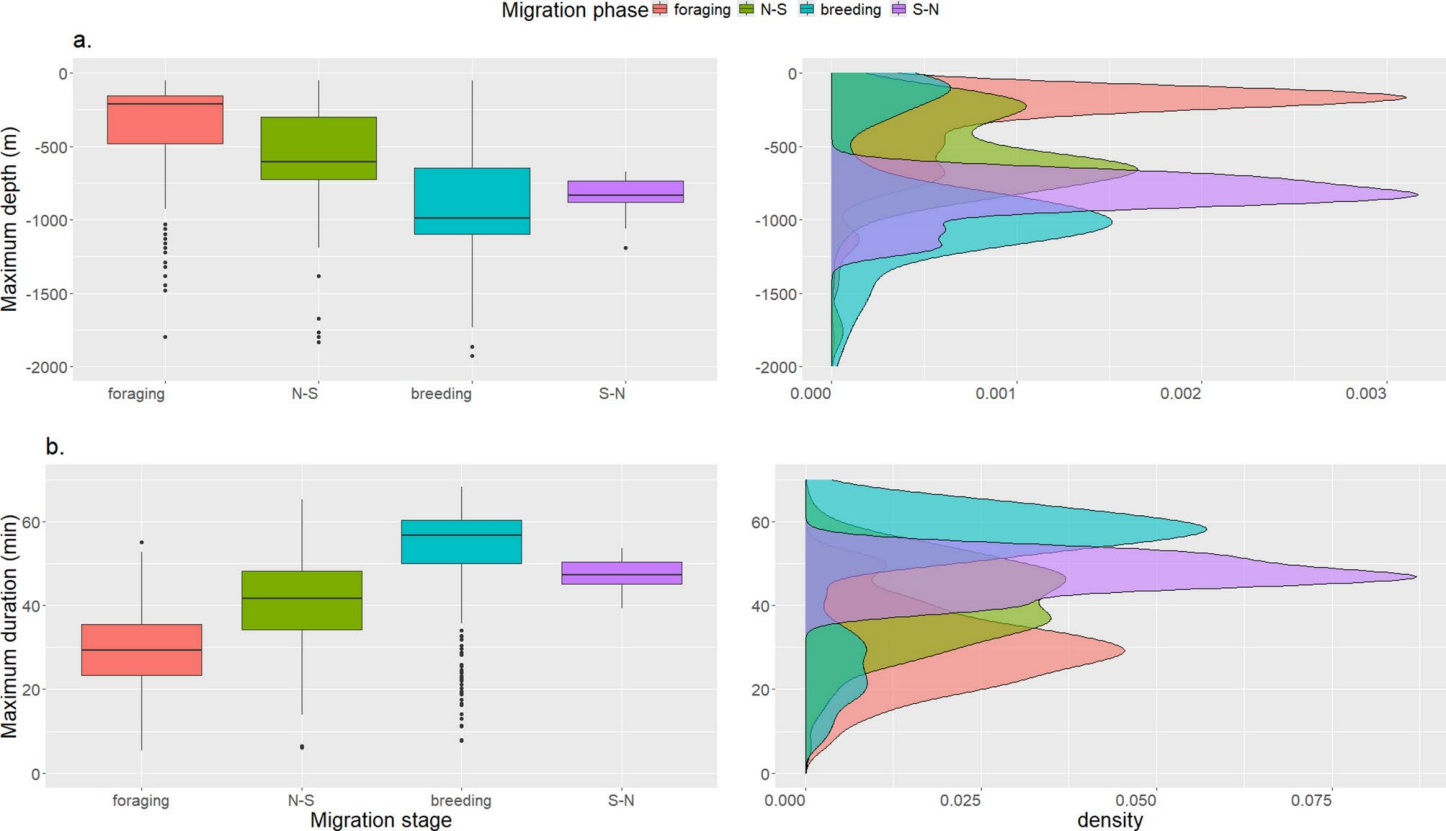
Maximum dive depth (**a**) and duration (**b**) from 1,054 dives of three adult male sperm whales equipped with satellite tags in Arctic Norway, 2020–2023 during their breeding migration cycle.

Dives were shorter (mean = 30 ± 10 min) and shallower (mean = 343 ± 283 m) in the foraging area compared to during transit phases (mean _N-S_ = 40 ± 10 min, mean _S–N_ = 47 ± 4 min—mean _N-S_ = 556 ± 296 m, mean _S–N_ = 847 ± 149 m) or in the breeding area (mean = 51 ± 15 min—mean = 849 ± 444 m; Fig. 7). LMEs confirmed that the migration phase was an important predictor of dive maximum depth (Δ AIC with null model = 105) and duration (Δ AIC with null model = 219). Post-hoc Tukey tests showed that all pairwise comparisons, between each of the behavioural phases, were significantly different except between the breeding area and S–N transit for the dive depth, and between the breeding area and S–N transit, and N-S and S–N transit for the dive duration (Table S1).

## Discussion

This study provides novel data on the behaviour of adult male sperm whales in the North Atlantic. Male sperm whales tagged in Arctic Norway migrated to low latitude breeding areas asynchronously throughout much of the year, suggesting that sperm whales do not have a specific breeding season. The breeding area occupied by the 12 migratory individuals in this study spanned across an enormous region that encompassed more than 10 million km^2^ in the tropical and temperate North Atlantic.

Despite the asynchrony in the timing of migration, all the migrating animals behaved very similarly during the transit parts of the migration, especially the N-S part. They travelled rapidly in a consistent south-westerly direction for an average of 40 (± 11) days to reach the breeding areas, at latitudes below 45°N. Speed, direction, move persistence and rate of latitudinal change were very similar across individuals. The highest estimated swim speeds occurred during the N-S transit when speeds averaged 4.7 km h^−1^. This horizontal travel speed is similar to what has been recorded for adult male sperm whales on their southbound trips both in the Northwest Atlantic and the Northeast Pacific^22–24^. It is also remarkably similar to travel speeds documented for humpback whales (*Megaptera novaeangliae*, 5.76 km h^−1^^41^) and fin whales (*Balaenoptera physalus*, 4.7 km h^−1^^42^) during their southbound migrations from the same general areas of the Northeast Atlantic Ocean. These baleen whales have annual cycles with quite strict phenologies because they must match their movements to the occurrence of seasonally available resources (accessible zooplankton pulses at high latitudes and seasonally fixed gatherings of mates at low latitudes). Male sperm whales do not operate under such seasonal constraints because they appear to be able to feed year-round at high latitudes and they do not have a seasonally restricted breeding season. Thus, their high N-S transit speed likely reflects a strong motivation to reach groups of females and concomitantly minimize time spent in transit through areas with low prey densities.

Once in the breeding grounds males showed more variable behaviour. They remained within the breeding area for periods of time ranging from 51 to 106 d (76 ± 22 days) and they travelled over a wide longitudinal range. None of the males spent any extended periods in coastal areas where sperm whales are often observed (like the Azores, Madeira, or the Caribbean arc) but rather looped through a vast area of open ocean. One male did do several loops south then north again without any distinct stopping in waters around the Azores. The average horizonal speed in the breeding area dropped from the N-S transit speed (4.7 km/h to 3.4 km/h) and move persistence became characteristic of area restricted search. Behavioural studies from sperm whale breeding grounds show that adult males generally roam between groups of females, spending only a few hours up to a few days with a particular group^43^. However, before the present study very little was known about the distribution of adult sperm whale males in the breeding areas in the North Atlantic, except those places where they occur relatively close to shore such as the east coast of the Americas and the Caribbean islands in the west, and in the Azores in the east^44,45^.

The distance from the start of migration to the furthest points on their paths ranged from 3,993–7,951 km, which is similar to what has been found for fin whales (about 5,000 km^42^), but somewhat shorter than distances reported for a female humpback whale (about 9,000 km^41^); both species in these studies were tagged in Arctic Norway. The cumulative distances for the two sperm whales that completed the entire cycles were 16,332 km and 17,669 km, respectively, which is closer to the entire breeding cycle recorded for the humpback whale mentioned above, which was some 18,300 km^41^.

Whether male sperm whales forage during the migration or in the breeding area remains unclear. But some individuals in this study did occasionally slow down during the transit phases and dive to depths greater than 1000 m, suggesting that they did some foraging. The deepest dive recorded in this study (1,927 m) took place in the breeding area, which further suggests some feeding occurs in this area. More research is needed to clarify this issue.

The average tracking period for the animals that did not migrate was shorter on average than for those that did (80 d vs 165 d), however there were also long data streams for some of the animals that remained in the north (up to 277 d). Conversely, some short tracking records were associated with migrating animals (44-60-61 and 72 days). We assume that all the “non-migrating” animals in our study do in fact migrate, but perhaps not necessarily on an annual basis.

The migrants in this study showed no clear seasonality with regards to when they initiated their migration towards their southern breeding ground. This finding is similar to what was found for migrating adult male sperm whale from Alaska^24^. Migration in animals generally evolves when some vital resources in their life are spatially and temporally separated, and often, as in the case of the adult male sperm whales in the present study, likely because locations of foraging opportunities are spatially separated from environments suitable for calves. Departures of males from the north in this study encompassed 10 months and only involved 12 individuals, supporting the belief that there is no clear seasonality in this event, but rather that individuals operate according to their own rhythms, likely some endogenous cues such as body condition. In comparison, baleen whales generally move to high latitudes during summers at a set time and often along traditional routes to take advantage of the rich, seasonal occurrence of production along ice edges and oceanographic fronts. In contrast, it is likely that there is comparatively little seasonality in the occurrence and availability of the squid and fish that the sperm whales feed on in the Northeast Atlantic^46–48^ and it is known that females in estrous are available year-round in the lower latitudes of the North Atlantic region^2,49^.

Female sperm whales and younger animals of both sexes generally spend their entire annual cycles at lower latitudes and obviously find enough food without migrating. Our migrating adult males dive at least sporadically to great depths at low latitudes, potentially feeding. So, why do adult males not just stay permanently in these lower latitude waters if they have year-around access to receptive females and food? When discussing migrations in baleen whales Corkeron and Connor^50^ suggested that one reason might be an “evolutionary holdover” where animals migrate because their ancestors did so. Whaling for sperm whales, first during the open boat whaling mainly with sailing ships during eighteenth and nineteenth centuries and later industrial whaling in the 1950s–1980s, dramatically reduced the global number of these whales^3^. The latter industrial whaling mainly targeted adult males^51^ and an estimated 39,000 sperm whales were removed from the North Atlantic Ocean during the twentieth century^52^. Since tropical and temperate waters are generally less productive than polar waters, the evolution of the migration pattern in sperm whales might have been a density-dependent necessity from when sperm whale numbers were much higher. There is little evidence for adult male sperm whales engaging in physical combat among themselves or for them defending females and young^1,51^. Males generally avoid each other and roam around more or less independently in breeding areas, although rare aggressive interactions have been observed^1^. Observations of interactions between mature males and females suggest that female mate choice may play an important role for male success in breeding^53^, and in this context size of the male might play a significant role, putting an evolutionary premium on males for growing fast and reaching a large size, which polar feeding might facilitate. Sperm whale clicks contain evenly spaced pulses where the inter-pulse interval is proportional to the size of the spermaceti organ (found in the head of sperm whales^54^). These acoustic displays can thus advertise size for potentially interested females and act as a deterrent for potential male competitors^8^. The clicks are audible to other whales at a distance of 60 km^8^ and may explain why adult male sperm whales are rarely observed close together at the breeding grounds^53^.

The degree of sociality of male sperm whales outside the breeding season is poorly known. But there is evidence that males aggregate in feeding areas^55,56^ and given that some individuals form long term associations^57^ it is unlikely to be solely due to prey densities. The males that travelled south in association in this study presumably did so by choice in a vast open ocean area. It is the norm to have loose clusters of males at high latitudes rather than randomly distributed individuals^56^. This aspect of the biology of sperm whales warrants further research.

There is a significant amount of data on sperm whale distribution from historic whaling, the most comprehensive source being Townsend^58^. This author went through logbooks from various whaling vessels from the years 1721 to 1920 and plotted catch positions for 36,909 sperm whales (all sex and age groups) in two maps covering the seasons from April to September and October to March, respectively. These catches were from all the worlds’ oceans but occurred mainly between 40°S and 40°N. When the tracks from our tagged whales are superimposed on Townsend’s^58^ map for the relevant season, we see that our whales visited many of these historical offshore whaling grounds (Fig. S5).

In a management context, tracking studies are useful for informing policy and management authorities about key habitats and species’ movements and behaviours, enabling relevant decisions regarding conservation. Knowledge about distribution of breeding areas and corridors is a key element in this respect. Most of the breeding area confirmed in the present study is in international waters. There is an “Agreement on Marine Biodiversity of Areas beyond National Jurisdiction”^59^, but this agreement is not yet specific enough to aid with species conservation. However, the extremely large spatial distribution of the sperm whales’ breeding area indirectly makes them robust to stochastic events unless they are very large scale, and the cessation of whaling and the relatively low ship traffic densities over most of the offshore breeding area are positive elements that might allow for the recovery of sperm whales in the North Atlantic Ocean. In the concluding remarks of a recently published review on sperm whale reproductive strategies^60^ it was stated that: “One gap in our understanding of sperm whales’ lives is the long-distance movements of mature males between feeding and breeding grounds”. This study’s results are at least a step in reducing the size of this gap, providing new data and insightful methodologies that will support further studies of these cosmopolitan giants.

## Supplementary Information

Supplementary Information.

## Data Availability

The datasets analysed during the current study are available at: https://zenodo.org/records/13879843. Also corresponding author can be contacted for access to the data.
