# Supplementary material for: Migration to breeding areas by male sperm whales Physeter macrocephalus from the Northeast Atlantic Arctic
Source: Sci Rep. 2025 Mar 6;15:7861. doi: 10.1038/s41598-025-91266-8 (PMC11885442; doi:10.1038/s41598-025-91266-8)
Supplement: Supplementary file 1 — Supplementary Information. [file 41598_2025_91266_MOESM1_ESM.docx]

**Supplementary material**

**Supplementary figure captions:**

**Figure S1**: Distribution of the maximum distance from the tagging location for 26 adult male sperm whales instrumented with satellite tags in Arctic Norway, 2020-2023 (a). Boxplot of the total tracking duration for the sperm whales that migrated from their northern feeding area (N=12) and those that did not migrate (N=14) during the tracking period (b).

**Figure S2**: Modelled tracks for 26 sperm whales instrumented with satellite tags in Arctic Norway, 2020-2023. Track from non-migrating individuals (N=14), a) and migrating individuals (N=12, b). The blue polygon represents the resident polygon based on the 100 % maximum convex polygon from tracking data for the non-migrating individuals. The maps were generated in Esri ArcMap v10.8.1, https://desktop.arcgis.com/en/arcmap/index.html.

**Figure S3.** Travel speeds for male adult sperm whales (N=12) tagged in Arctic Norway, 2020-2023, during their breeding migrations. Red dashed vertical line represents the start of the southward migration; green dashed line indicates the arrival at the breeding area; pink dashed line indicates the departure northward from the breeding area; light blue dashed line indicates the return to the northern feeding area. The black lines represent the daily speed, and the thick red line represents the smoothed daily speed.

**Figure S4.** Distribution of bearings along the north-south migratory path of adult male sperm whales instrumented with satellite tags in Arctic Norway, 2020-2023.

**Figure S5.** Maps of catch positions for 36,909 sperm whales (all sex and age groups) extracted from logbooks from various whaling vessels from the years 1721 to 1920 by Townsend (1935). Upper panel covers the season April - September and lower panel October - March. Tracks from adult male sperm whales equipped with satellite tags in Arctic Norway, 2020-2023, are superimposed on the two panels according to the date these animals entered these areas. The various coloured circles in the maps represents different months of the year (Upper panel: April–green open circle, Ma –green filled circle, June–blue open circle, July-blue filled circle, August-red open circle, September-red filled circle. Lower panel: October-black open circle, November-black filled circle, December-dark blue open circle, January-dark blue filled circle, February-orange open circle, March-orange filled circle).


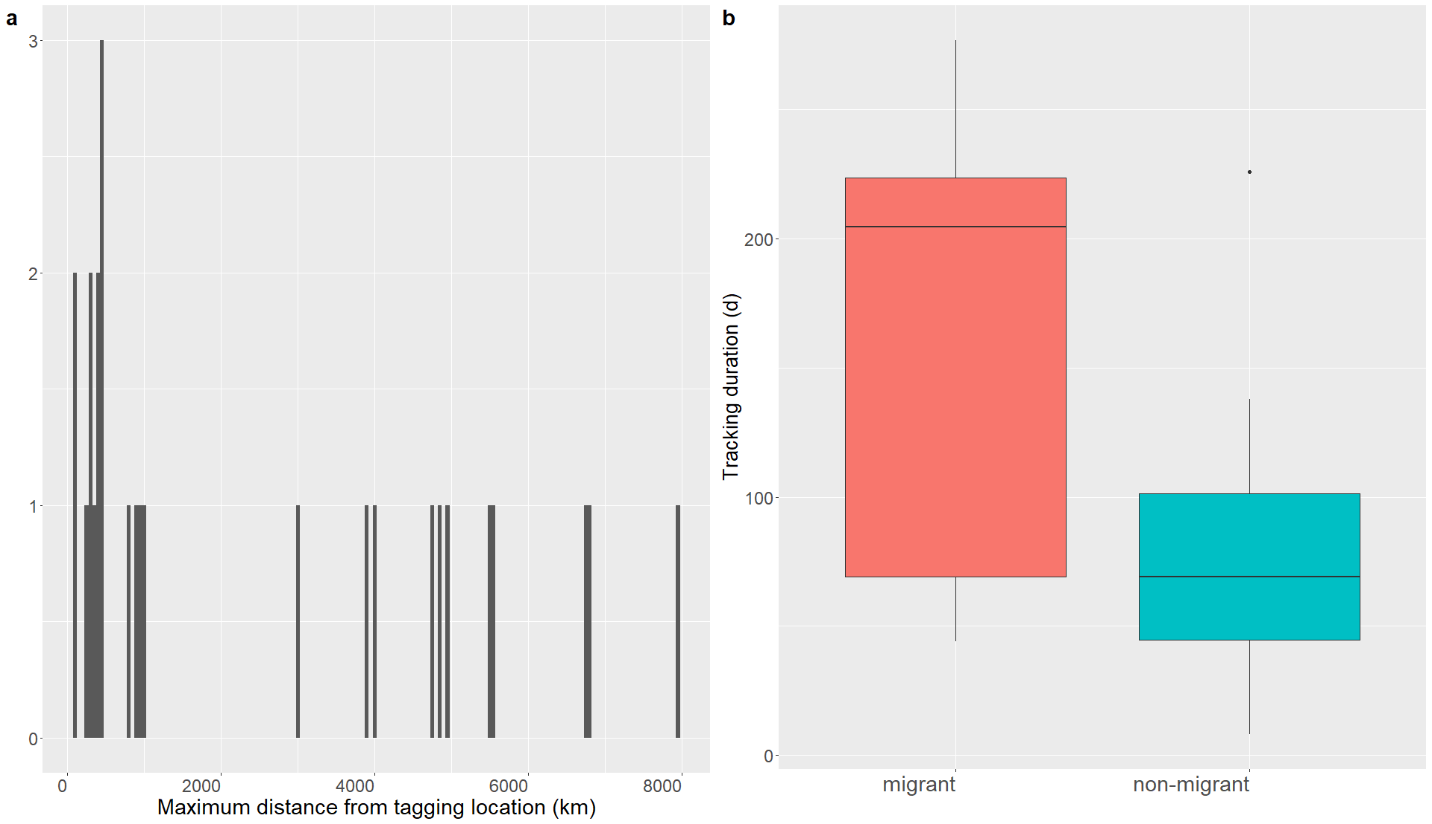


Figure S1


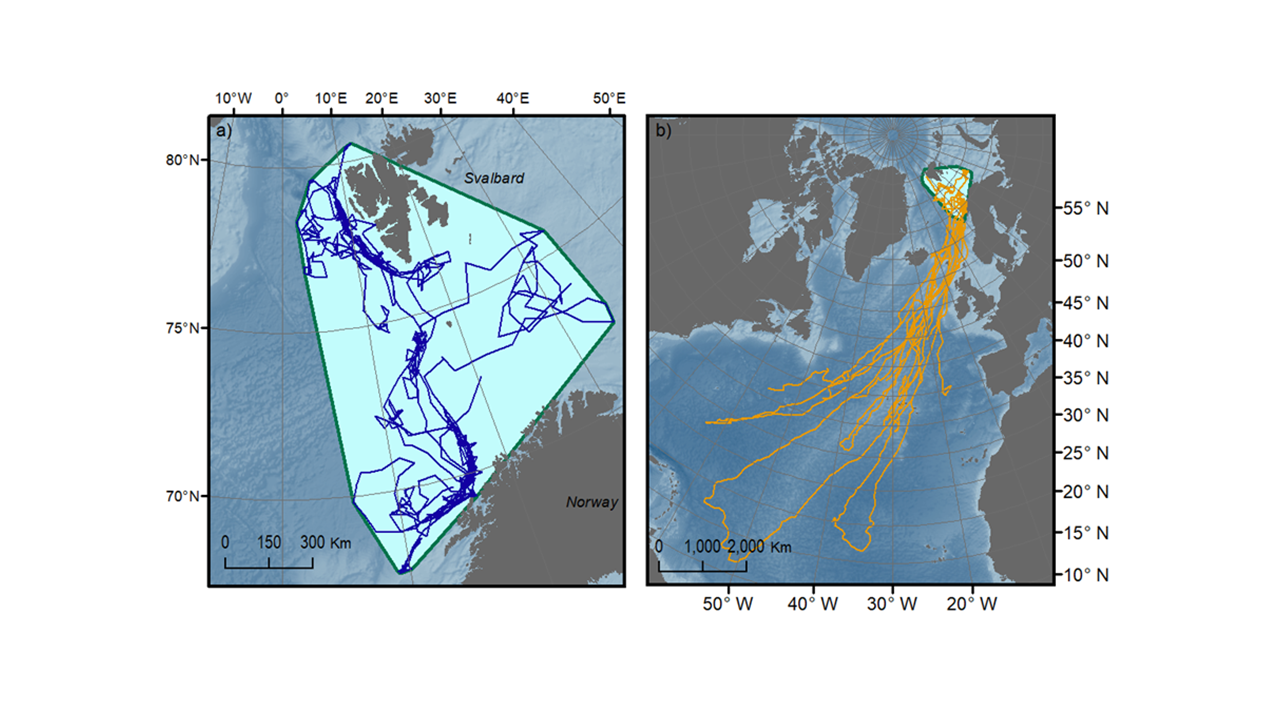


Figure S2

**
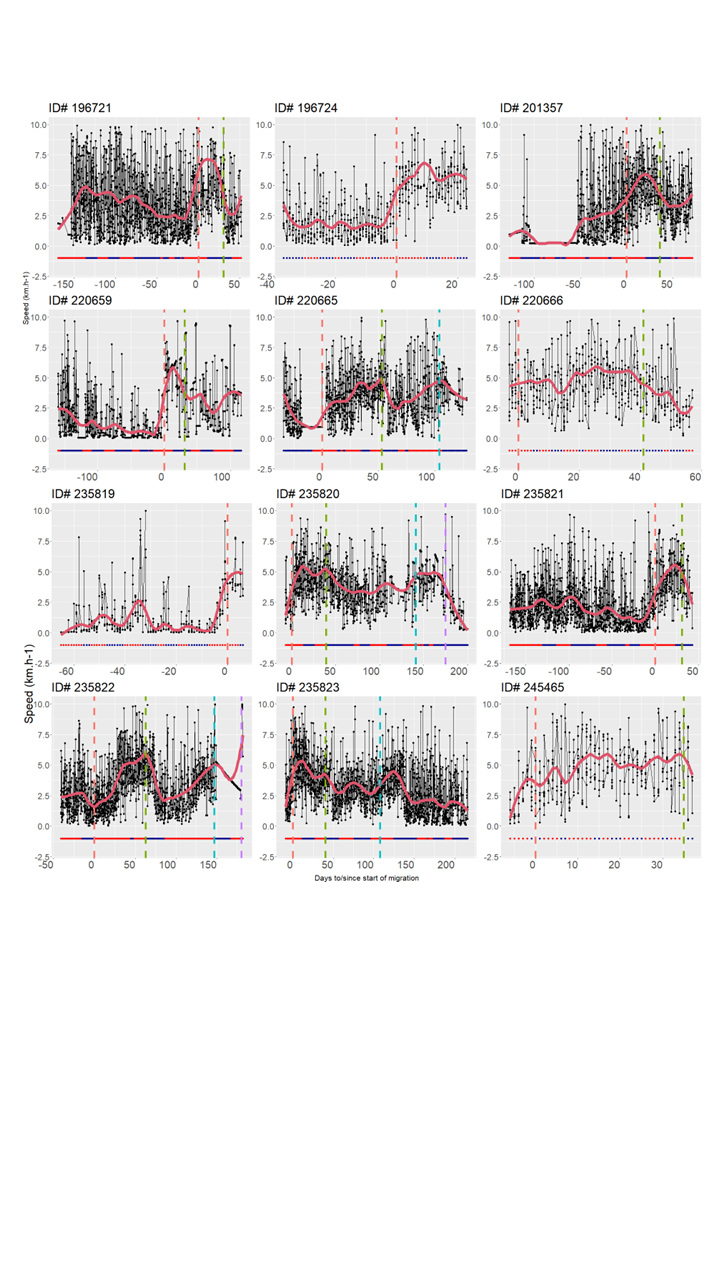
**

Figure S3

**
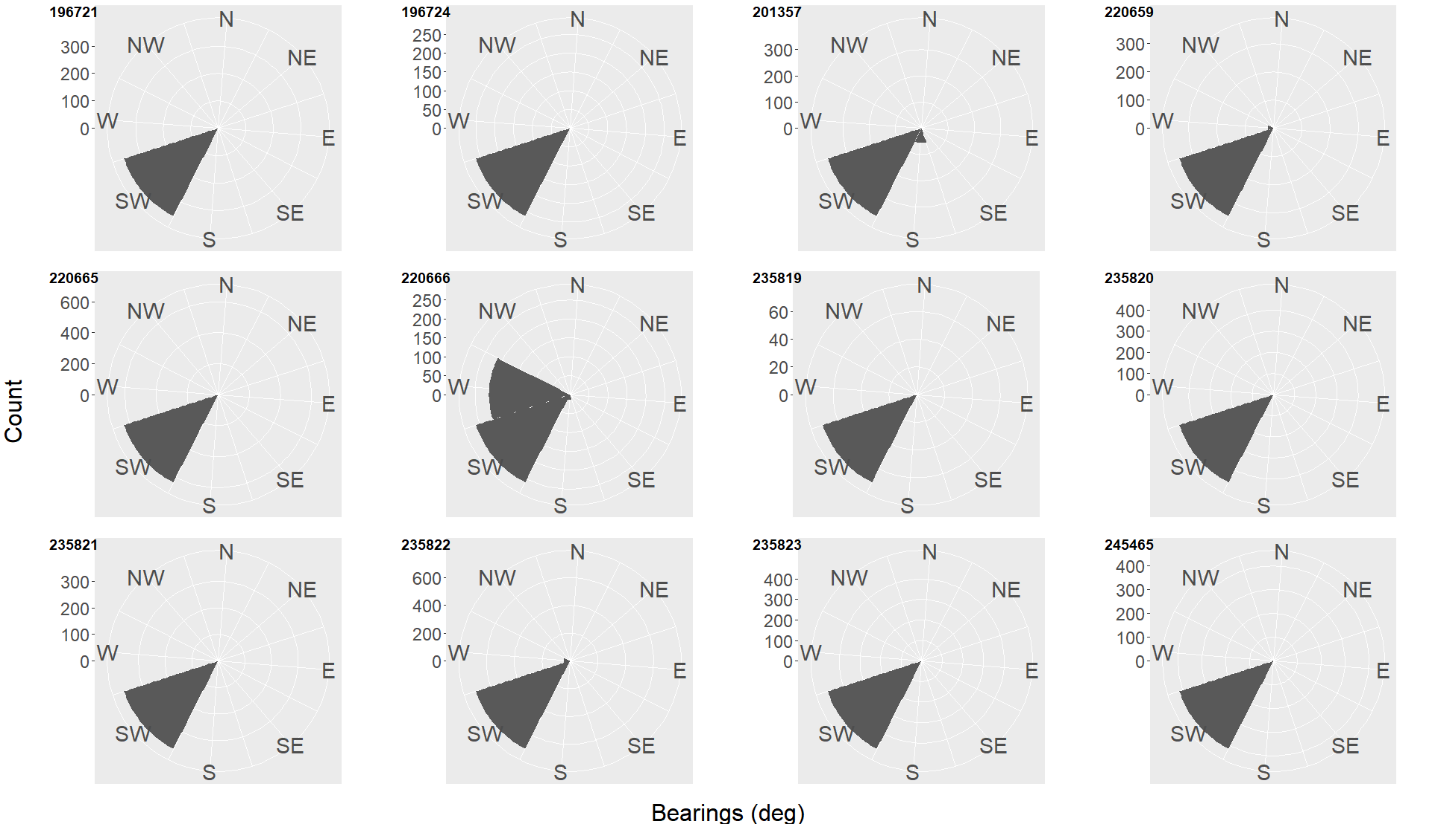
**

Figure S4

**
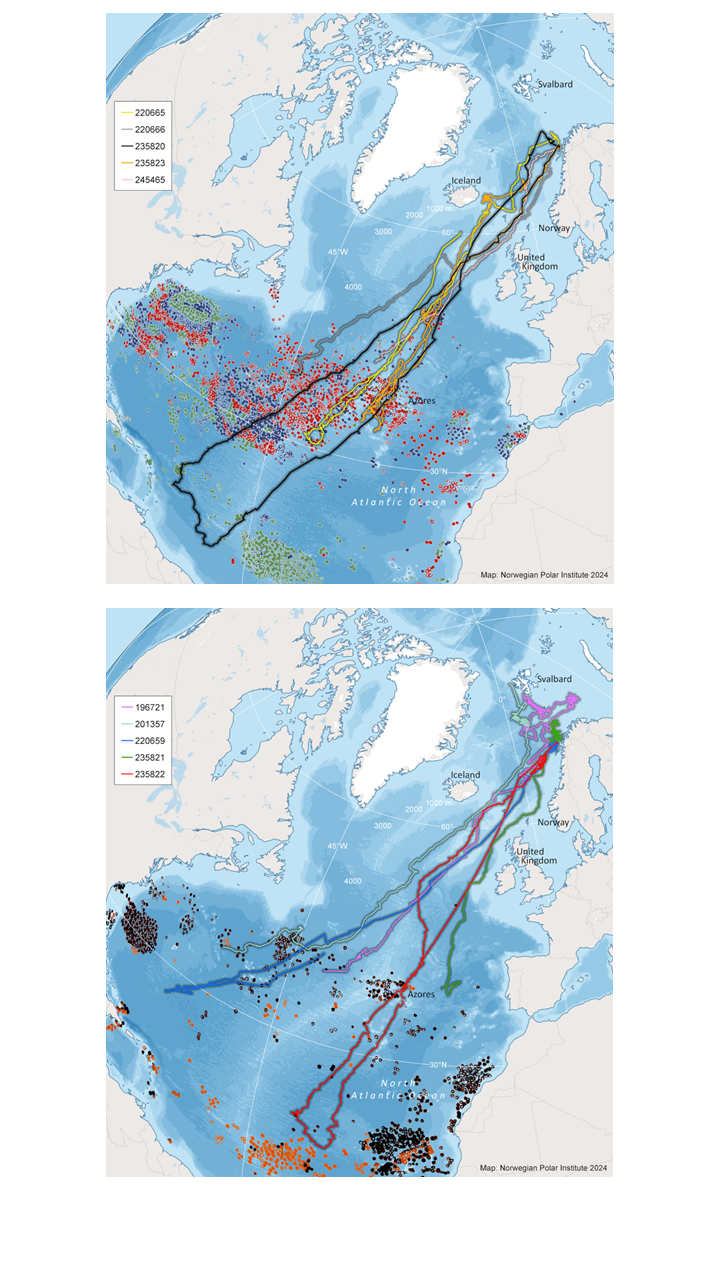
**

Figure S5

**Table S1**. Results of the pairwise post-hoc Tukey test to assess the difference in dive depth and duration between the migration phases for adult male sperm whales instrumented with satellite tags in Arctic Norway.

| **Dive depth** | **Pairwise** | **t.ratio** | **p.value** |
| --- | --- | --- | --- |
|  | residential vs N-S | -6.108 | <.0001 |
|  | residential vs breeding | -10.462 | <.0001 |
|  | residential vs S-N | -4.928 | <.0001 |
|  | N-S vs breeding | -5.648 | <.0001 |
|  | N-S vs S-N | -3.198 | 0.0078 |
|  | breeding vs S-N | -1.182 | 0.6382 |
|  |  |  |  |
| **Dive duration** | **Pairwise** | **t.ratio** | **p.value** |
|  | residential vs N-S | -9.71 | <.0001 |
|  | residential vs breeding | -15.959 | <.0001 |
|  | residential vs S-N | -4.311 | 0.0001 |
|  | N-S vs breeding | -8.279 | <.0001 |
|  | N-S vs S-N | -1.535 | 0.4171 |
|  | breeding vs S-N | 1.417 | 0.4892 |
